# A Novel Murine Model of Acute Laryngeal Injury After Intubation

**DOI:** 10.1002/lary.70244

**Published:** 2025-10-30

**Authors:** Ruth J. Davis, Hannah Kreuser, Tadeas Lunga, Ryan E. Schaub, Susan L. Thibeault

**Affiliations:** ^1^ Department of Surgery, Division of Otolaryngology—Head and Neck Surgery University of Wisconsin‐Madison School of Medicine and Public Health Madison Wisconsin USA

**Keywords:** acute laryngeal injury, glottic stenosis, mouse model

## Abstract

**Objective(s):**

Acute laryngeal injury (ALgI) occurs in over 50% of patients after intubation and mechanical ventilation and is associated with significantly worse voice, breathing, and swallowing outcomes. Currently, there are no small animal models for the study of ALgI and its progression to glottic stenosis. The objective of this study was to develop and validate a novel murine model of ALgI.

**Methods:**

Thirty adult C57BL/6 mice underwent chemomechanical injury to the posterior glottis, and 16 control mice did not undergo injury. Glottic injury was performed under endoscopic guidance using a bleomycin‐dipped wire brush. Mice underwent repeat endoscopy at 14‐ or 21‐days following injury, and the maximal interarytenoid angle during respiration was quantified using ImageJ to evaluate glottic mobility. Histologic and gene expression analyses were performed on larynges from each group.

**Results:**

The interarytenoid angle of injured mice was significantly reduced compared to controls at both 14 (35.3° vs. 68.0°, *p* = 0.016) and 21 days post‐injury (34.5° vs. 68.0°, *p* < 0.001). There was a significant increase in posterior glottic thickness in injured compared to control mice at 21 (132.5 vs. 53.9, *p* < 0.001) but not 14 days post‐injury (90.4 vs. 53.9, *p* = 0.1535). Trichrome staining and RT‐qPCR demonstrated collagen upregulation in the posterior glottis of injured mice.

**Conclusion:**

Chemomechanical injury to the posterior glottis produces a novel murine model of ALgI. This safe, reliable, and feasible model lays the foundation for future translational study of ALgI and its progression to glottic stenosis.

**Level of Evidence:**

N/A animal study.

## Graphical abstract

This study describes a novel murine model of acute laryngeal injury (ALgI) after intubation. Chemomechanical injury was performed to the posterior glottis of adult mice using a bleomycin‐dipped wire brush under endoscopic guidance, resulting in reduced glottic mobility, increased posterior glottic thickness, and increased collagen expression. This safe, reliable, and feasible model lays the foundation for future translational study of ALgI and its progression to glottic stenosis.

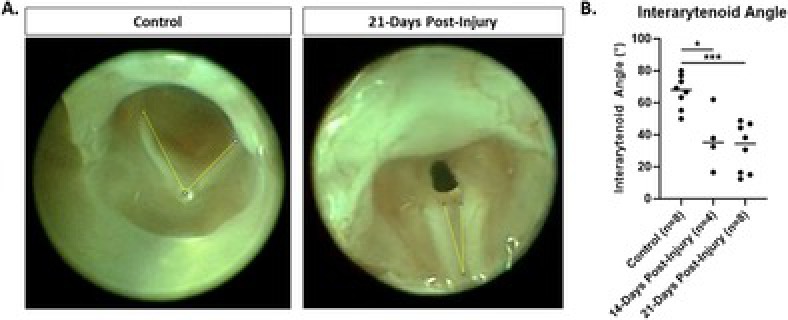

## 1 Introduction

Acute laryngeal injury (ALgI) occurs in over 50% of patients after intubation and mechanical ventilation [1, 2]. It is associated with significantly worse voice, breathing, and swallowing months after extubation [1, 2, 3]. ALgI results from endotracheal tube pressure on the posterior glottis, leading to ischemic mucosal injury that progresses to glottic scar and debilitating phonatory insufficiency or life‐threatening narrowing of the glottic airway.

Current treatment for glottic stenosis consists of destructive surgical interventions (i.e., cordotomy), open airway reconstruction, or permanent tracheostomy, all of which sacrifice vocal quality for airway patency [4, 5]. Mature glottic stenosis is particularly refractory to treatment, with 42% of patients remaining tracheostomy dependent despite an average of 11 surgeries per patient, in pursuit of decannulation [6].

The molecular study of intubation‐related airway injury has primarily focused on tracheal rather than glottic stenosis, resulting in a critical gap in understanding the biological pathophysiology of post‐intubation glottic scar [7, 8, 9, 10, 11]. Histologic analysis demonstrates fibrosis, inflammation, and cricoarytenoid joint fusion [12, 13, 14, 15, 16, 17], however the pathophysiology of glottic stenosis has not been evaluated on a cellular level. Post‐intubation tracheal stenosis can be modeled in animals ranging in size from mice [18] to sheep [19]. This range in animal models facilitates a variety of experiments studying the pathophysiology of disease in addition to the efficacy of medical and surgical interventions in the preclinical setting.

Given the complexity of glottic tissue architecture and coordinated motion required for phonation, respiration, and deglutition, even minor glottic scars can have devastating functional consequences [20]. When the glottis is rendered immobile by scar, surgical intervention is rarely able to restore motion, and a trade‐off must be made between static glottic opening to enable respiration or static glottic closure to enable phonation [21]. Currently, there is no ideal animal model for the study of ALgI and its progression to posterior glottic stenosis (PGS). Published animal models of PGS include canine [14, 17], porcine [15, 16, 22], and rabbit [12, 13] models; however larger animal models are limited by expense and lack of available reagents (such as primers, antibodies, etc.) or genetically modified animals. In this study, we describe a novel murine model of ALgI and its progression to PGS.

## 2 Materials and Methods

### 2.1 Experimental Design

This study was approved by the University of Wisconsin—Madison Animal Care and Use Committee (IACUC # M006756). Forty‐six adult C57BL/6 mice were used in this study. Mice were purchased from Jackson Laboratory (strain #000664, Bar Harbor, ME, USA) and bred by the University of Wisconsin Biomedical Research Model Services. Equal numbers of male and female mice were used in the control and 21‐day post‐injury groups. Only males were used at the 14‐day timepoint.

### 2.2 Murine Laryngoscopy

Mice underwent laryngoscopy as previously described [23]. Mice were anesthetized with 5% inhaled isoflurane in an induction chamber, then transferred to a platform where maintenance 2% inhaled isoflurane was delivered via a custom nose cone with silicone O‐rings secured to the maxillary incisors and mandible to maintain mouth opening as previously described [23, 24]. Laryngeal endoscopy was performed using a 1.9 mm diameter 30° Hopkins endoscope (64301 BA, Karl Storz, El Segundo, CA, USA) within a 2.5‐mm‐diameter operating sheath with a 3 Fr (~1 mm) working channel (61029 D, Karl Storz), xenon light source (Kay Elemetrics Xenon 7150, KayPENTAX Inc., Lincoln Park, NJ, USA), and lightweight color camera (Tricam NTSC Camera System, Karl Storz). Endoscopic images were recorded on a high‐definition video capture system (SDC HD, Stryker Corporation, Kalamazoo, MI, USA).

### 2.3 Posterior Glottic Injury

**FIGURE 1:**
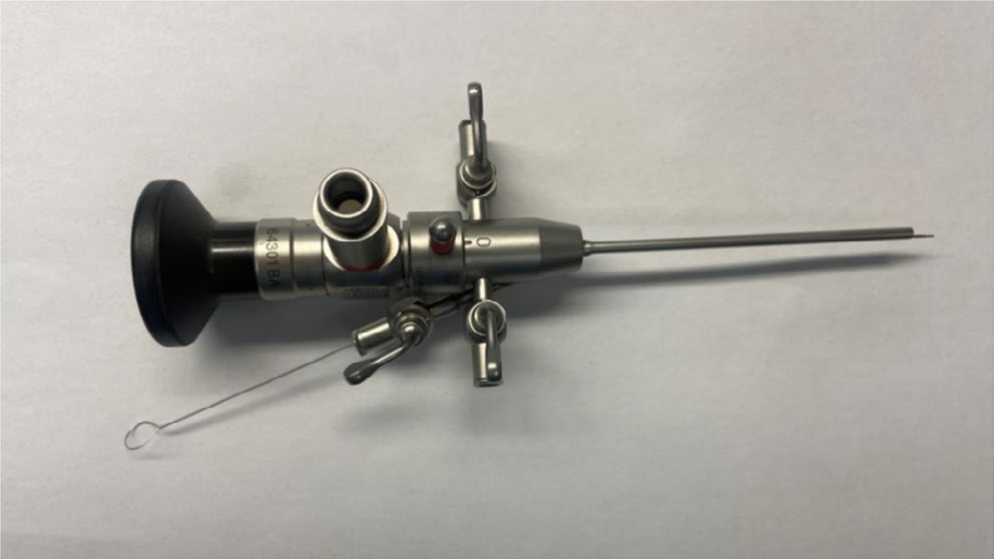
Experimental setup. Operating sheath with 30° endoscope and custom 0.028‐in. diameter stainless steel wire brush inserted through the working channel. Custom micro‐spiral brush specifications: 0.028″ OD × 1/8″ brush part × 7″ overall length, 0.003″ stainless steel brush wire, 0.012″ stainless steel base wire (approximately 0.020″ stem diameter), 1/16″ tip length with Ø 1/4″ ring handle.

**FIGURE 2:**
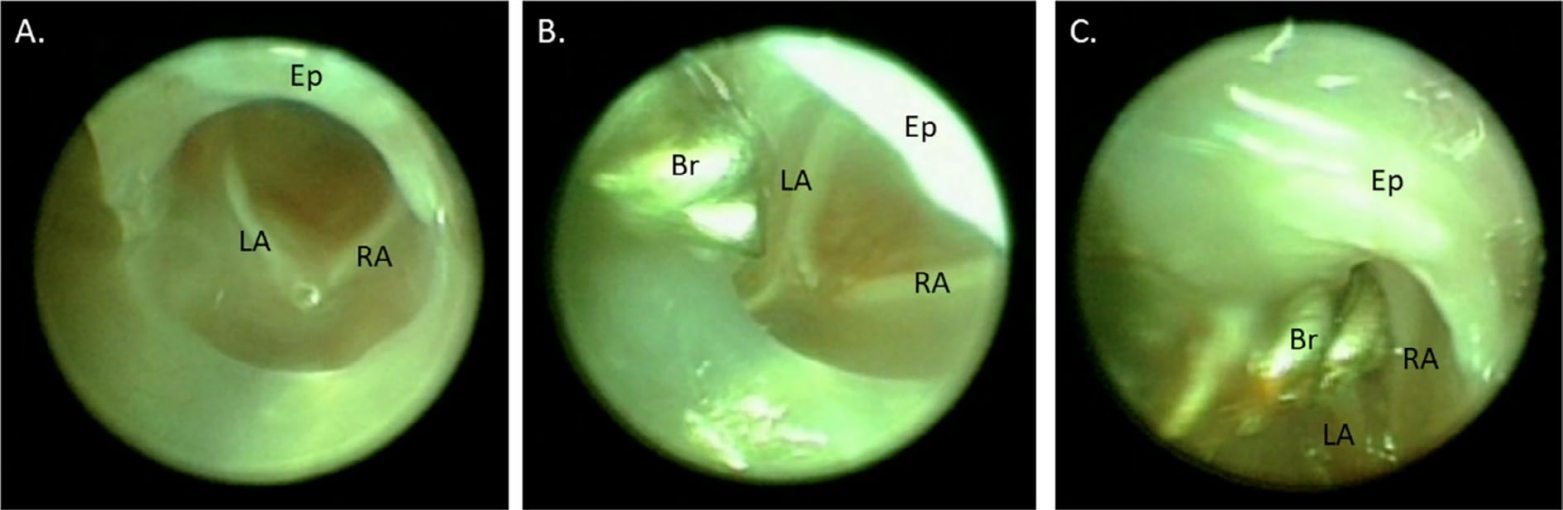
Injury procedure. (A) Endoscopic view of the murine larynx prior to injury, (B) brush advanced through the working channel of the endoscope sheath towards the larynx, (C) brush passing between the arytenoids to injure the posterior glottis. Br, brush; Ep, epiglottis; LA, left arytenoid; RA, right arytenoid.

A 0.028‐in. outer diameter custom steel wire brush (Gordon Brush Mfg. Co. Inc., City of Industry, CA, USA) was dipped into a solution of 1 U/mL bleomycin (NorthStar Rx LLC, Memphis, TN, USA). This was then passed through the working channel of the operating sheath and used under direct visualization to abrade the posterior laryngeal epithelium with five passes of the brush (Figures 1 and 2, Video 1). The brush and endoscope were then removed, and the entire process was repeated after dipping the brush into the bleomycin solution a second time. Control mice underwent no injury.

**VIDEO 1:** Injury procedure. Endoscopic video of the laryngeal injury procedure. Video content can be viewed at https://onlinelibrary.wiley.com/doi/10.1002/lary.70244.

### 2.4 Animal Health

The health of all mice was assessed immediately after emergence from anesthesia, a second time within 24 h of the procedure, and again on a weekly basis. Animals were euthanized at BCS ≤ 2 or if respiratory distress was observed.

### 2.5 Endoscopic Assessment

**FIGURE 3:**
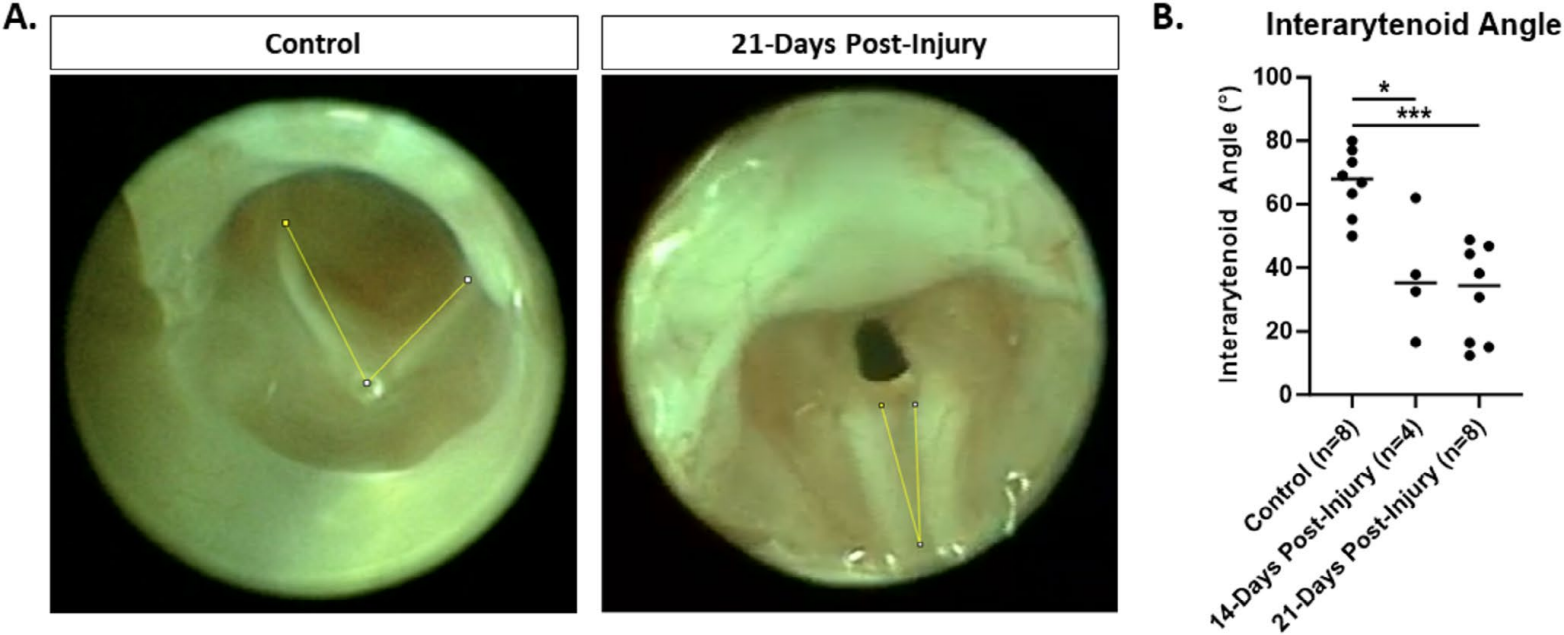
Maximal glottic opening. (A) Endoscopic images at maximal abduction demonstrate reduced glottic opening 21‐days post‐injury compared to uninjured control. Yellow lines depict measurement of interarytenoid angle using Image J software. (B) There is a significant reduction in maximal interarytenoid angle at both 14‐ and 21‐days post‐injury. *p < 0.025, ***p < 0.001.

At the designated time points, 20 mice were again anesthetized with inhaled isoflurane with maintenance of spontaneous respiration. Laryngoscopy was repeated as described above and videos were recorded. Mice were then euthanized with CO_2_ immediately following this procedure. Videos were played back frame by frame for analysis and screen capture of three instances of maximal arytenoid abduction with respiration by a blinded reviewer. ImageJ [25] was used to measure the interarytenoid angle on each image, and an average was calculated for each animal (Figure 3). The reviewer was blinded to the treatment group and timepoint. A second blinded reviewer re‐evaluated 20% of the images for inter‐rater reliability.

### 2.6 Tissue Collection and Histology

Following endoscopy and euthanasia as described above, larynges were dissected free from surrounding tissues using a dissection microscope. Larynges were fixed in 4% PFA, then each specimen was embedded in paraffin. Five‐micron‐thick sections were cut in a transverse plane through the larynx taking care to remain parallel with the plane of the vocal folds for a consistent angle of sectioning. Slides were stained with hematoxylin–eosin (H&E) or Masson's Trichrome. Slides were imaged on a Nikon Eclipse Ti2 inverted microscope with NIS Elements software (Nikon, Tokyo, Japan). Histologic sections incorporating bilateral cricoarytenoid joints, bilateral thyroarytenoid muscles and the anterior commissure were selected for analysis to ensure a consistent angle of sectioning, and these were photographed at ×10 magnification. ImageJ [25] was used to measure subepithelial thickness in the midline posterior glottis on H&E slides by a reviewer who was blinded to the treatment group and timepoint. A second blinded reviewer re‐evaluated 20% of the slides for inter‐rater reliability.

### 2.7 Gene Expression Analysis

Larynges were collected from euthanized mice as above. An additional 8 uninjured mice and 16 mice at 21 days post‐injury were included for gene expression analysis. The cricoarytenoid complex was further dissected from the remaining laryngeal tissue in PBS using the dissection microscope and placed in RNA*later* (Invitrogen, Waltham, MA, USA) at 4°C and moved to −20°C after 24 h for storage. RNA was extracted using the RNeasy Plus Mini Kit (Qiagen, Hilden, Germany), with tissue disrupted using a mortar and pestle followed by homogenization using a QIAshredder homogenizer (Qiagen). RNA was quantified using a NanoDrop 1000 Spectrophotometer (Thermo Fisher Scientific, Waltham, MA, USA). All cDNA was synthesized from 500 ng of RNA using GoScript Reverse Transcription Mix, Random Primers (Promega, Madison, WI) in 20 μL reactions following the manufacturer's instructions. Transcripts were quantified using PowerUp SYBR Green Master Mix (Applied Biosystems, Waltham, MA, USA) on a 7500 Fast Real‐Time PCR System machine (Applied Biosystems). Triplicate 20 μL qPCR technical replicates for each sample contained 0.8 μL of cDNA (from 20 ng of RNA) and 500 nM of both forward and reverse primers. Transcript levels were normalized to *ActB* (ΔC_*T*_). Changes in gene expression were calculated using the 2^−ΔΔC^_*T*_ method, subtracting the ΔC_*T*_ of the control samples from the injured samples. The following primers were used in this study: *ActB*, forward: 5′‐AGAGGGAAATCGTGCGTGAC, reverse: 5′‐CCACAGGATTCCATACCCAAG; *Col1a1*, forward: 5′‐GGAGAGAGCATGACCGATGG, reverse: 5′‐AAGTTCCGGTGTGACTCGTG.

### 2.8 Statistical Analysis

Statistical analyses were performed using Prism software, version 10.1.2 (GraphPad Software Inc., Boston, MA). A nonparametric Mann–Whitney test was used to assess differences in interarytenoid angle and posterior glottic thickness between controls and the 14‐ and 21‐day post‐injury timepoints. A Wilcoxon matched pairs signed rank test was used to evaluate inter‐rater reliability of the interarytenoid angle and posterior glottic thickness measurements. A one sample Wilcoxon signed rank test was used to assess the significance of the gene expression fold change 21 days post‐injury compared to controls. A nonparametric Mann–Whitney test was used to evaluate differences by sex. A type I error rate (*α*) < 0.05 was considered statistically significant. A Bonferroni correction (*α*/*n*) was applied to adjust for multiple hypotheses (*n* = 2) when comparing control to the 14‐ and 21‐day post‐injury timepoints, setting the level of statistical significance at a *p* < 0.025 for these analyses.

## 3 Results

### 3.1 Adverse Events

There were two deaths out of 30 injured mice (6.7% mortality rate). One mouse demonstrated respiratory distress upon emergence from anesthesia and was euthanized immediately after the injury procedure. The second mouse was found dead 7 days later, and upon postmortem dissection was found to have purulence in the neck from a suspected pharyngeal perforation during injury.

### 3.2 Reduced Glottic Opening

The impact of chemomechanical laryngeal injury upon glottic mobility was evaluated by comparing the interarytenoid angle at maximal abduction 14 and 21 days after injury to uninjured control mice. The interarytenoid angle of injured mice was significantly reduced compared to controls at both 14 (median 35.3° vs. 68.0°, *p* = 0.016) and 21 days post‐injury (Figure 3; median 34.5° vs. 68.0°, *p* < 0.001). At 21 days post‐injury, there was no significant difference in interarytenoid angle between male and female mice (median 26.6° vs. 37.6°, *p* = 0.686). There was also no significant difference between the two reviewers for 20% of the images (median 43.6° vs. 43.0°, *p* = 0.875).

### 3.3 Increased Posterior Glottic Thickness

**FIGURE 4:**
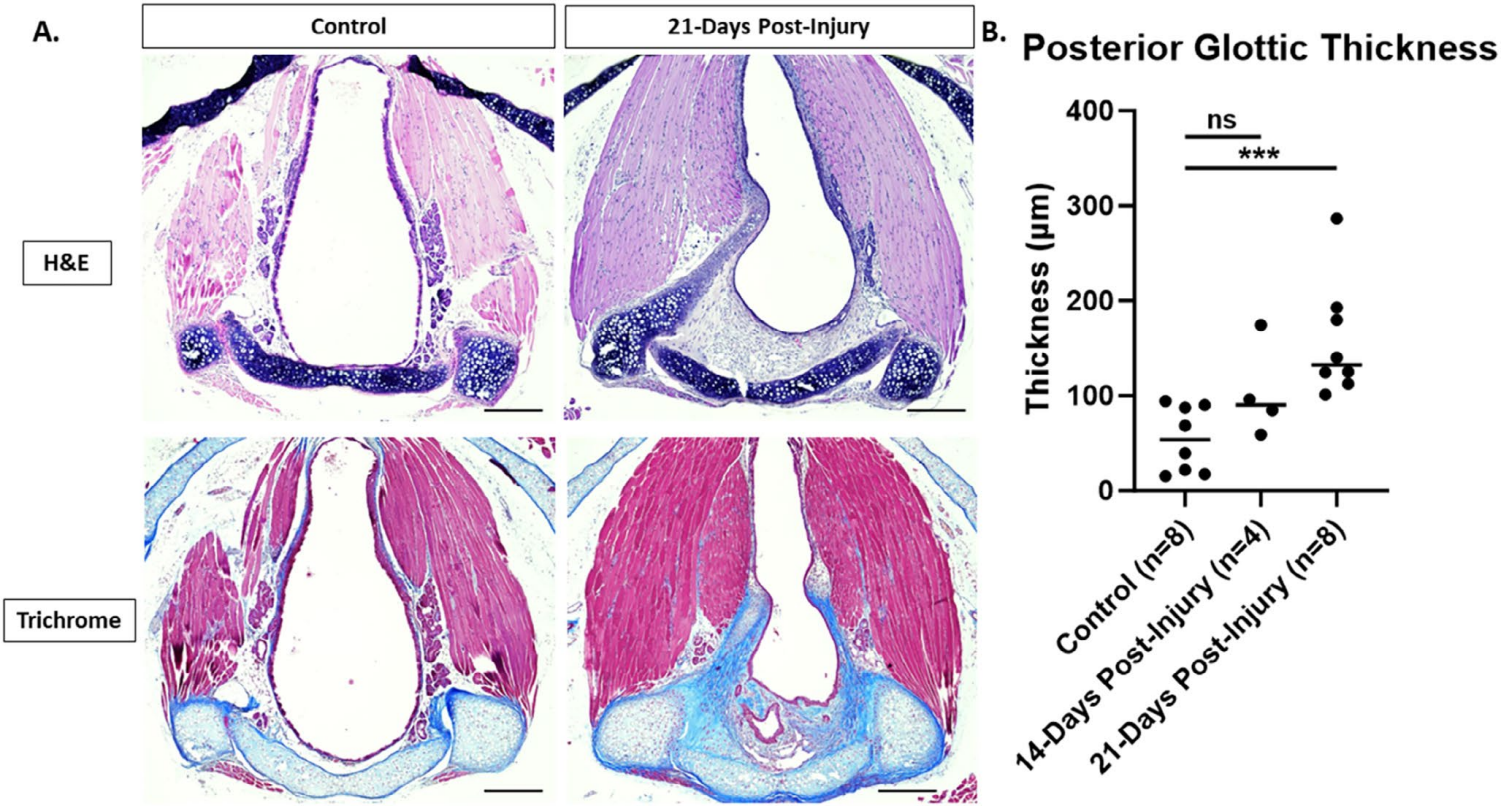
Histologic changes. (A) H&E and Masson's Trichrome stained transverse sections of control and 21‐day post‐injury larynges. (B) There is a significant increase in posterior glottic thickness at 21‐days but not 14‐days post‐injury. Images obtained at ×10 magnification, scale bar: 250 μm, ***p < 0.001.

Subepithelial thickness of the lamina propria in the posterior glottis was measured to evaluate the impact of chemomechanical laryngeal injury upon the development of glottic stenosis. There was a significant increase in posterior glottic thickness in injured compared to control mice at 21 (median 132.5 vs. 53.9 μm, *p* < 0.001) but not 14 days post‐injury (Figure 4; median 90.4 vs. 53.9 μm, *p* = 0.154). At 21 days post‐injury, there was no significant difference in posterior glottic thickness between male and female mice (median 146.2 vs. 132.5 μm, *p* = 0.886). There was no significant difference between the two reviewers for 20% of the slides (median 98.6 vs. 96.4 μm, *p* = 0.875).

### 3.4 Increased Extracellular Matrix

**FIGURE 5:**
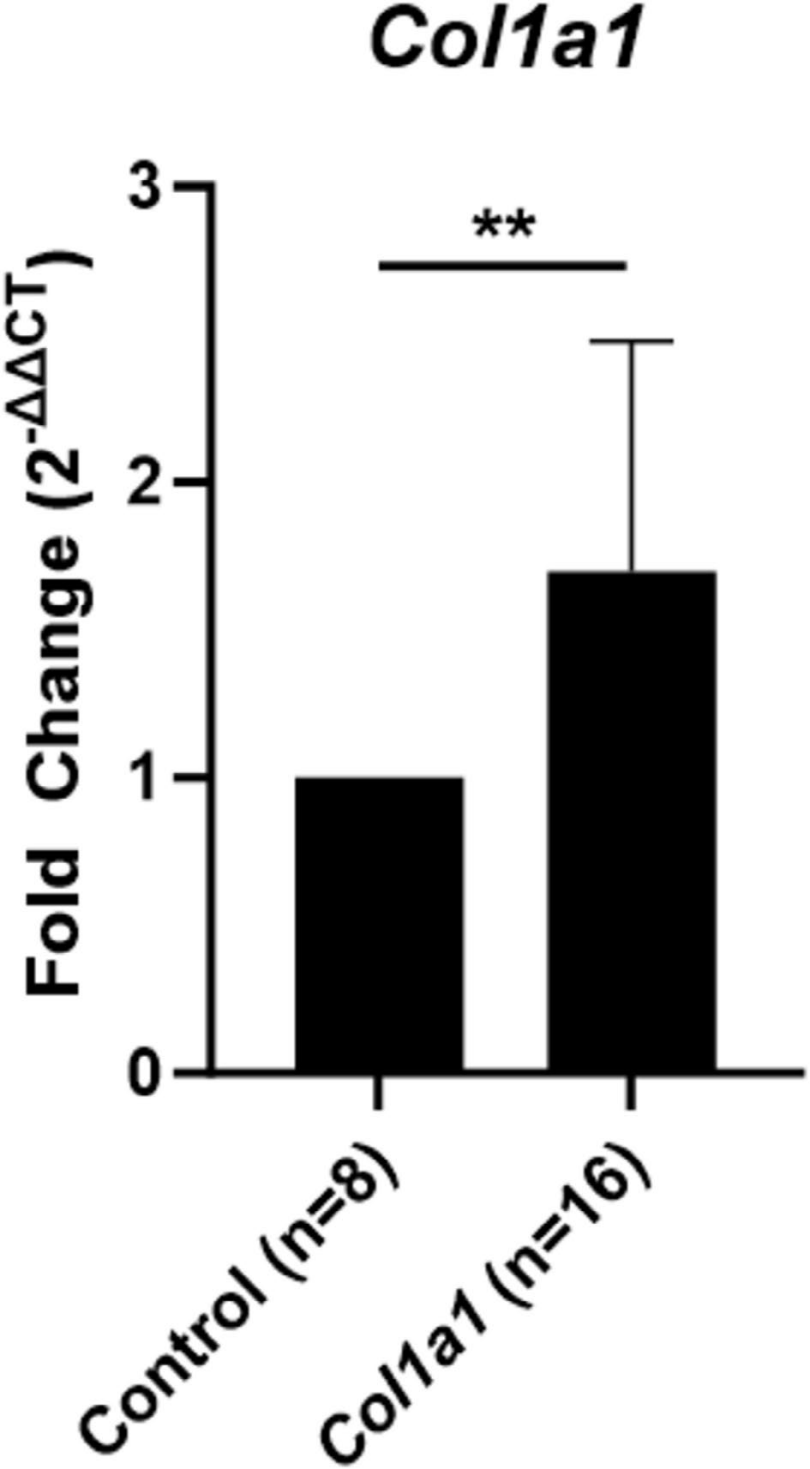
*Col1a1* expression. RT‐qPCR demonstrates significant upregulation of *Col1a1* gene expression at 21‐days following injury. **p < 0.01.

Collagen deposition was evaluated to assess glottic scar formation 21 days after laryngeal injury. Trichrome staining demonstrated blue collagen in the thickened submucosa of the posterior glottis 21 days following injury (Figure 4). RT‐qPCR was performed to quantify collagen alpha‐1(I) chain (*Col1a1*) gene expression changes following injury. A significant increase in *Col1a1* gene expression (Figure 5; median 1.37‐fold upregulation, *p* = 0.002) was observed 21 days after injury compared to uninjured controls. There was no significant difference in *Col1a1* gene expression between male and female mice 21 days after injury (1.91 vs. 1.25‐fold upregulation, *p* = 0.328).

## 4 Discussion

ALgI occurs in over 50% of patients after endotracheal intubation [1, 2] and is the precursor lesion to PGS and post‐intubation phonatory insufficiency, which are both exceedingly challenging to treat [6, 26]. There is currently no murine model of ALgI to facilitate preclinical study of the pathophysiology of ALgI and its progression to PGS or to evaluate potential therapeutic strategies.

Previously described canine models of post‐intubation laryngeal injury have included suturing of an endotracheal tube segment to the endolarynx for periods of 24 h to 84 days [14] or performing carbon dioxide (CO_2_) laser ablation of varying depths to the posterior glottis [17]. In rabbits, posterior glottic injury with either CO_2_ laser ablation [12] or cold instrument mucosal stripping of the posterior glottis [13] resulted in posterior glottic scar formation. In pigs, hypoxic ventilation and intubation resulted in ALgI on histologic assessment [15, 22], however mature scar has not been assessed following this terminal procedure. A rat model of 3‐ or 6‐h intubation with suction tubing resulted in mucosal thickening and gland hypertrophy 24 h later; however, it demonstrated mortality rates of 16.7% and 44.4%, respectively [27]. This mortality rate in the rat model raises concerns regarding survival of a similar procedure if performed in mice given the smaller size.

Therefore, to develop a novel murine model of ALgI, concepts from validated murine models of tracheal stenosis [18] and vocal fold injury were combined [23]. The use of endoscopic guidance to facilitate a focal injury to the posterior glottis limits nonspecific injury and associated edema that may contribute to increased mortality in other approaches to injure the small rodent airway [18, 27]. Chemomechanical injury with the bleomycin‐dipped brush was chosen as the mechanism to increase the reliability of scar formation compared to mechanical injury, alone [18]. Bleomycin is an antineoplastic antibiotic with fibrotic side effects, including pulmonary fibrosis. Endotracheal bleomycin is used extensively in animal models of pulmonary fibrosis [28], and has therefore been adapted for use in a murine model of tracheal stenosis [18] and now ALgI in the present study.

Murine models are valuable tools in the study of human disease given their decreased cost compared to larger animals, and the access to a wider range of commercially available reagents (such as primers, antibodies, etc.) and genetically modified animals. In addition to these advantages, the novel murine model described in this study provides a functional outcome measure (maximal glottic opening) in addition to traditional histologic and molecular outcomes to evaluate the impact of posterior glottic injury.

The measurement of the glottic angle at maximal abduction is well established in the literature as a means to quantify glottic opening. The anterior glottic angle can be reliably measured in humans on laryngoscopy with minimal interjudge variability [29]. When measured using the deep learning algorithm, Automated Glottic Action Tracking by artificial Intelligence (AGATI) [30], an anterior glottic angle less than 40.96° identified patients with bilateral vocal fold immobility with a sensitivity of 86% and specificity of 94% [30, 31]. In this study the mean AGA was 29.9° in patients with bilateral vocal fold immobility and 57.08° in normal patients without laryngeal pathology [31].

However, in mice visualization of the anterior commissure is challenging due to epiglottic prolapse (Figures 2 and 3) [32]. Therefore, tracking of arytenoid cartilage motion [32, 33] and measurement of the interarytenoid angle [34] have been used to quantify laryngeal motion after recurrent laryngeal nerve injury in mice. Similarly, our model takes advantage of the fact that unlike in humans, the murine arytenoids come together to form an angle at the posterior glottis during maximal abduction, forming a point that mirrors the anterior commissure. This angle can be quantified and just as in humans with bilateral vocal fold immobility, the angle is significantly decreased following posterior glottic injury in this model (Figure 4). The angle is not dependent upon the distance of the telescope from the glottis, which is difficult to control. Three measurements per mouse were averaged to account for variability in respiratory cycles.

Histologic and gene expression data confirm that at 21 days post‐injury, tissue remodeling and scar formation with deposition of type 1 collagen following glottic injury is associated with this reduced glottic opening (Figures 4 and 5). Type 1 collagen is an extracellular matrix component that is deposited in excess in scar tissue [35]. Upregulation in type 1 collagen is a consistent feature of human airway stenosis [36, 37] and animal models of tracheal stenosis [19, 38].

Limitations of this model include the use of a procedural technique that is more prone to variation than a genetic or chemical model. Location or depth of injury may be inconsistent from one animal to another or from one proceduralist to another, and the procedure requires a learning curve to become facile with this precise technique. Variability in the degree of stenosis was observed following injury (Figures 3 and 4), which may be due to unrecognized variability in the severity of injury or variability in wound healing. For these reasons, it is recommended that one experienced proceduralist perform all injuries when comparing injury rates among different conditions. Future efforts to optimize the laryngeal view during injury, such as incorporating a micromanipulator to hold the endoscope [32], may improve the consistency of injury in this model.

In addition, the acute chemomechanical injury in this model may not fully recapitulate chronic pressure injury resulting from long‐term intubation and mechanical ventilation. Long‐term intubation of mice is likely not feasible as a survival procedure given the small airway size; however bleomycin is a well‐established pro‐fibrotic adjunct utilized extensively in mouse models of pulmonary and tracheal fibrosis [18, 39, 40]. Similar to intubation injury to the airway, bleomycin results in epithelial injury, release of reactive oxygen species, pro‐inflammatory and pro‐fibrotic cytokines, and excessive extracellular matrix production [41]. Although fundamentally different from chronic pressure injury, chemomechanical injury to the trachea with a bleomycin‐coated brush has demonstrated inflammatory and fibrotic parallels to post‐intubation tracheal stenosis [10, 18]. Future studies may incorporate serial brush injury without bleomycin to more closely model the chronic injury that occurs with intubation.

## 5 Conclusion

Chemomechanical injury to the posterior glottis produces a novel murine model of ALgI. This model is safe, reliable, feasible, and incorporates a functional outcome of glottic mobility. This model lays the foundation for future translational study of the pathophysiology of ALgI and the prevention of its progression to glottic stenosis.

## Conflicts of Interest

The authors declare no conflicts of interest.

## Data Availability

The data that support the findings of this study are available from the corresponding author upon reasonable request.
